# Identification of an AgS_2_ Complex on Ag(110)

**DOI:** 10.1038/s41598-019-56275-4

**Published:** 2019-12-27

**Authors:** Peter M. Spurgeon, Da-Jiang Liu, Junepyo Oh, Yousoo Kim, Patricia A. Thiel

**Affiliations:** 1 Department of Chemistry, Iowa State University, Ames, Iowa 50011 USA; 2 RIKEN Surface and Interface Science Laboratory, RIKEN, Wako, Saitama 351-0198 Japan; 3 Ames Laboratory of the USDOE, Ames, Iowa 50011 USA; 4 Department of Materials Science and Engineering, Iowa State University, Ames, Iowa 50011 USA

**Keywords:** Chemistry, Nanoscience and technology, Physics

## Abstract

Adsorbed sulfur has been investigated on the Ag(110) surface at two different coverages, 0.02 and 0.25 monolayers. At the lower coverage, only sulfur adatoms are present. At the higher coverage, there are additional bright features which we identify as linear, independent AgS_2_ complexes. This identification is based upon density functional theory (DFT) and its comparison with experimental observations including bias dependence and separation between complexes. DFT also predicts the absence of AgS_2_ complexes at low coverage, and the development of AgS_2_ complexes around a coverage of 0.25 monolayers of sulfur, as is experimentally observed. To our knowledge, this is the first example of an isolated linear sulfur-metal-sulfur complex.

## Introduction

Silver is an important industrial catalyst that plays a role in reactions such as epoxidation of ethylene^1–4^, dehydrogenation of methanol^5–8^, and oxidation of CO^9–11^. Because of this, the interactions of oxygen with silver substrates or supported silver nanoparticles have been studied extensively^12–19^. While sulfur has not received as much attention as oxygen, many studies have examined sulfur-containing molecules on silver surfaces due to the widely known tarnishing of silver by sulfur containing vapors in the atmosphere^20–22^. Also, sulfur is a known poisoning agent for many metal catalysts^23,24^, so it is important to understand the effects that adsorbed sulfur can have on metal nanoparticles.

Sulfur is also known as an important adsorbate in terms of affecting the stability of nanoparticles, i.e. increasing their susceptibility to coarsening. Previous coarsening studies of adsorbed sulfur on coinage metal surfaces have shown that even trace amounts of sulfur lead to a dramatic destabilization of metal nanoislands on the surface^25–27^. There is mounting evidence that supports the reason for the accelerated destabilization of the metal islands as being the formation of mobile surface mass carriers^28,29^. The mass carriers are proposed to be metal sulfur complexes. For the coinage metal surfaces, metal-sulfur complexes were observed with low-temperature STM on Cu(111)^28^, Ag(111)^29^, and Au(100)^30^, with structures proposed for each of the complexes. A common motif observed in these metal-sulfur complex structures is a linear sulfur-metal-sulfur unit, although that unit does not exist—or at least, has not been observed—independently from the larger complexes. In this paper, we will show for the first time (to our knowledge) that it can be isolated and observed directly, when sulfur is adsorbed on the Ag(110) surface.

A related system, sulfur on Cu(110), has been studied at sub-monolayer sulfur coverages. It was found that S adatoms coexist with possible Cu_x_S_y_ clusters^31^. No detailed structures of these clusters have been proposed and the clusters were imaged at 77 K, leading to the clusters appearing streaky or fuzzy in STM images due to the clusters being semi-mobile on the surface. In another related system, it has been demonstrated using low-temperature STM, that oxygen on Ag(110) can produce a variety of features^32,33^. Two of these features contain a linear O-Ag-O structure. One feature is a zigzag chain of -O-Ag-O-Ag-O- aligned along the [1 −1 0] direction^32^. The zigzag chain is composed of silver atoms within a row of the substrate being partially displaced vertically and the oxygen occupying three-fold hollow sites along the substrate rows. The chain can be regarded as oxygen atoms densely decorating a pre-existing row of Ag atoms. The second feature is an isolated, linear O-Ag-O unit, where the central Ag atom is embedded in (but slightly displaced from) a pre-existing row of Ag atoms^33^. It was proposed that the embedded complex can detach from its lateral surroundings and become “free,” hence aiding in the creation of surface vacancies. These “free” AgO_2_ complexes may serve as the building blocks of the Ag(110)-O-(2 × 1) added row reconstruction^33^. However, they were not observed directly. In this paper we will show that there are some similarities but also major differences between AgO_2_ complexes and AgS_2_ complexes on this surface.

This paper is organized as follows. Section 2 describes the experimental and computational methods. Section 3 presents the results, and Section 4 is a discussion. Auxiliary information is given in the Supplemental Information.

## Methods

### Experimental details

More detailed description of experimental conditions are provided elsewhere^34^. To summarize, a single Ag(110) crystal was cleaned via Ar^+^ sputtering and annealing cycles. S_2_ (gas) was generated with the sample held at room temperature by an electrochemical evaporator that has been described in previous studies^28,30,34–36^. Sulfur coverage (*θ*_*S*_), in units of monolayers, was determined by counting protrusions in STM images in a given area (individual S adatoms associated with small protrusions and the brighter and larger protrusions with 2 S), and taking the ratio of S atoms to the number of Ag atoms in the Ag(110) surface plane.

The primary experimental technique was low temperature STM, imaging at 5 K. The XY (in-plane) piezoelectrics were calibrated against the p(1 × 1) of the clean Ag(110) substrate; consequently, lengths measured along the [1 −1 0] direction were multiplied by a factor of 1.1. There was no significant distortion in the [1 0 0] direction. All dimensions are reported accordingly, though the STM images themselves remain unadjusted. The Z (vertical) calibration was checked using step heights, and agreement with the expected bulk value was within 1.4%. All tunneling voltages, *V*_*S*_, are given as sample bias.

### Computational details

#### Energetics

Energetics were assessed by methods similar to those employed in earlier work^30,37–39^. Briefly, DFT calculations were performed using the plane-wave based VASP code^40^ with standard PAW potentials^41^ optimized for the PBE method^42,43^ that were distributed with versions 5.2 and higher. The energy cutoff was 280 eV. Gamma centered *k*-point grids that correspond approximately to (24 × 17 × 1) for primitive (1 × 1) cells were used. All atoms in a slab were allowed to relax except the bottom layer. Energies were averaged over values for slabs in a range of thicknesses, *L* = 7 to 12 (in layers), to mitigate quantum size effects^44^ which are strong on Ag(110)^45^. Error bars in graphs, and parentheses in numerical values, show estimated uncertainties due to different slab thicknesses unless otherwise stated.

The relative stability of species that (potentially) incorporate both S and Ag, such as complexes and reconstructions, must reflect the energetic cost of providing both. The chemical potential per S atom, *µ*_*S*_, serves this purpose, where *µ*_*S*_ is defined as:

1 $${{\mu }}_{S}=[E({{\rm{Ag}}}_{{\rm{m}}}{{\rm{S}}}_{{\rm{n}}}+{\rm{slab}})-E({\rm{slab}})-{\rm{m}}{{\mu }}_{Ag}]/n-E({{\rm{S}}}_{2,{\rm{g}}})/2$$

here *E* is energy, while *µ*_*Ag*_ is the chemical potential of Ag in the bulk metal (at 0 K), which equals the bulk cohesive energy and serves as the energy reference point for the metal. If bulk and surface are equilibrated, *µ*_*Ag*_ also equals the binding energy of a Ag atom at a kink site^46^. The energy of the triplet state of the gas-phase dimer, *E*(S_2,g_), serves as the energy reference point for S. Choosing gas-phase S_2_ reduces the significant ambiguity and error that would arise in the calculation of the self-energy of an atom using DFT. Since a positive *µ*_S_ thus defined means the system is unstable towards associative desorption of S_2_, it is also more physically relevant. The integers *m* and *n* are the number of Ag and S atoms in the complex, respectively. When *m* = 0, *µ*_*S*_ is simply the adsorption energy of the S adatom.

#### Simulating STM images

Elsewhere^34^, we have described the modified Tersoff-Hamann method^47,48^ used to generate simulated STM images using DFT. For purposes of comparison with experimental data, two main parameters are the tunneling current *I*, and the bias voltage *V*_*S*_, which together fix the gap between tip and sample. In the simulations, *I* can only be expressed in arbitrary units, but a low current of *I* = 1 × 10^−3^ a.u. corresponds to a realistic gap of 0.5–0.8 nm, whereas larger *I* correspond to smaller, less realistic gaps. In turn, achieving low *I* places certain demands on the calculations. The vacuum between slabs must be large and the energy cutoff must be high, to ensure appropriate exponential decay of electron density into vacuum. In this work we use 600 eV and 2.1 nm for image simulation, respectively. For image simulation, we also average over various values of *L* = 7 to 12.

## Experimental and Computational Results

### Sulfur adatoms at very low coverage

**Figure 1:**
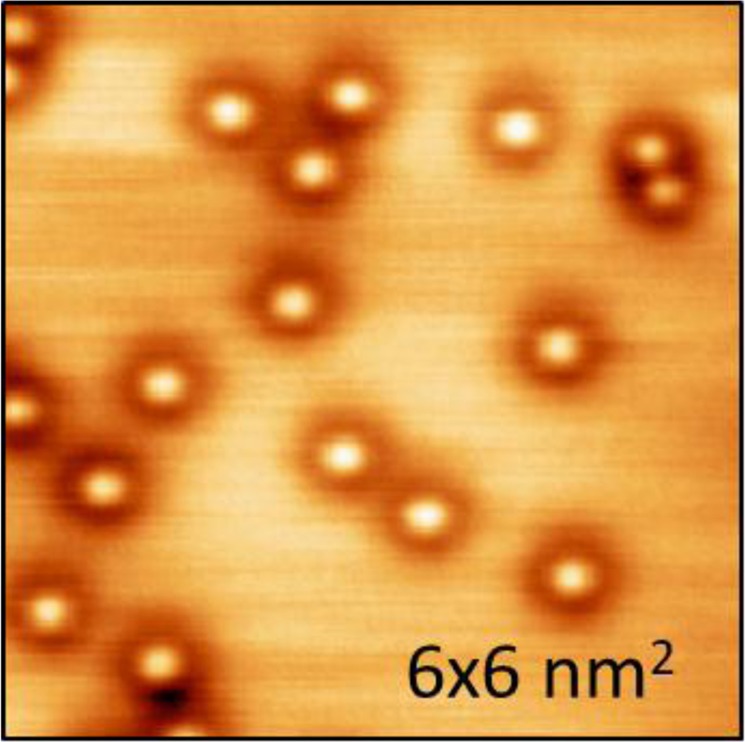
STM image of 0.02 ML S on Ag(110). *I* = 1.0 nA, *V*_*S*_ = −1.2 V.

**Figure 2:**
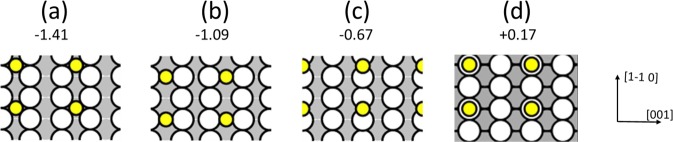
Models of S adatoms in p(2 × 2) superstructures at 4 different high-symmetry sites on Ag(110), with associated chemical potentials *µ*_*S*_ (in eV) from DFT. White large circles represent Ag atoms in the surface plane, yellow small circles show S adatoms. (**a**) Two-fold hollow site. (**b**) Long bridge site. (**c**) Short bridge site. (**d**) On-top site.

At low sulfur coverage, only sulfur adatoms are observed. They are illustrated in Fig. 1 for *θ*_*S*_ = 0.02 ML, where each bright spot surrounded by a dark ring (sombrero) corresponds to a sulfur atom. The full-width at half-maximum (FWHM) of the central protrusion, at negative *V*_*S*_, is 0.31 ± 0.04 nm, which compares favorably with sizes of single sulfur adatoms measured under comparable conditions on Ag(100)^34^, Cu(100)^35^, and Au(111)^36^, where the FWHM ranges from 0.34 to 0.38 nm. Furthermore, as has been reported previously^34^, the central protrusion in these features disappears with increasing *V*_*S*_, leaving only a dark depression at sufficiently positive *V*_*S*_. The reason for this was clarified from DFT^34^; in short, the change in appearance is caused by different rates of change in through-surface and through-adsorbate conductances^49^ as *V*_*S*_ increases. This transition from protrusion to depression with increasing *V*_*S*_ is thus expected for sulfur adatoms on Ag(110), from DFT. Finally, the preferred adsorption site of the sulfur adatom is the two-fold hollow site, in the trough, as illustrated in Fig. 2a. DFT shows that this is favored over other high-symmetry sites, based upon the values of chemical potential *μ*_*S*_ given in Fig. 2. Sulfur is known to occupy this site on other structurally-similar surfaces: Cu(110)^50^, Ni(110)^51,52^, and Rh(110)^53^. Later, we will show that DFT also predicts that sulfur adatoms are more stable than sulfur complexes at low coverages *θ*_*S*_ $\lesssim$ 0.25 ML, hence reinforcing the conclusion that these sombreros are sulfur adatoms.

### Coexisting bright features and S adatoms at 0.25 ML

**Figure 3:**
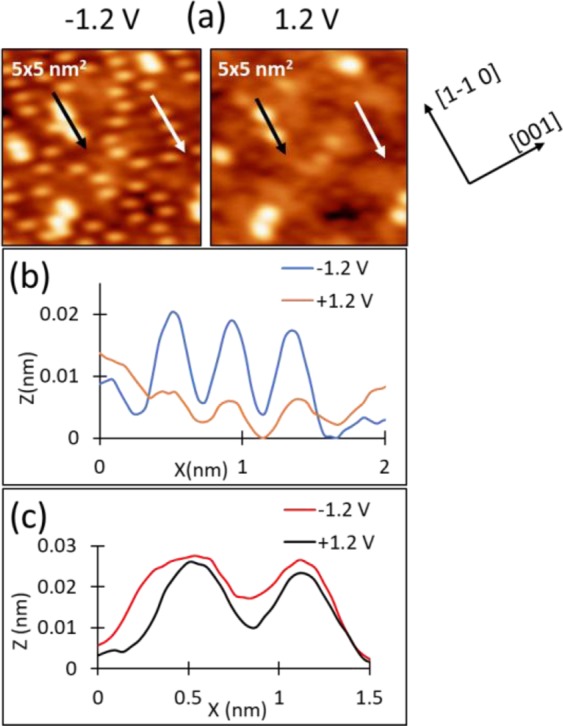
(**a**) STM images and corresponding line profiles of two types of features, at *θ*_*S*_ = 0.25, and *V*_*S*_ = −1.2 V and +1.2 V. The location of the line profiles for S adatoms correspond to the white arrows in each image, while the location of the line profiles for AgS_2_ complexes correspond to the black arrows in each image. (**b**) corresponds to the line profiles of S adatoms at two different values of *V*_*S*_, and (**c**) corresponds to the line profile of AgS_2_ complexes at two different values of *V*_*S*_. Tunneling current at both voltages is *I* = 0.9 nA. The S adatoms appear compressed in the [1 −1 0] direction, which is an experimental artifact.

At a higher sulfur coverage of 0.25 ML, two types of features coexist in the STM images, as shown in Fig. 3. The smaller and more prevalent species are identified as S adatoms based on the characteristics of the central protrusion at *V*_*S*_ = −1V. At this *V*_*S*_, the size of the protrusions matches the size of S adatoms identified at 0.02 ML. Unlike S adatoms at 0.02 ML S, where the adatoms switch from sombreros to depressions as *V*_*S*_ changes from negative to positive, at 0.25 ML the S adatoms stay as sombreros but with a reduction in height [Fig. 3b]. This is consistent with the prediction from DFT and previous observations with STM, showing that the bias-dependence weakens as *θ*_*S*_ increases^34^.

**Figure 4:**
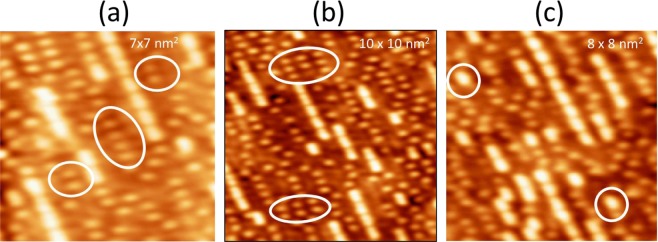
STM image showing two types of features, at *θ*_*S*_ = 0.25. The ovals in panel (a) show chains of S adatoms separated by 2a_1_. Ovals in panel (b) show regions with local p(2 × 2) order of S adatoms. Ovals in (**c**) show individual bright features, identified as AgS_2_ complexes. Tunneling conditions (*I, V*_*S*_) are: (**a**) 0.9 nA, −1.2 V; (**b**) 0.9 nA, −1.2 V; (**c**) 1.0 nA, −1.0 V.

Sulfur adatoms exhibit local ordering at *θ*_*S*_ = 0.25 ML. Approximately half the S adatoms exist in small chains that are 2–5 S atoms long in the [1 −1 0] direction [Fig. 4a, ovals]. The spacing between S adatoms in a chain is 0.44 ± 0.02 nm, which is 2a_1_ (2x larger than the experimental unit cell length in the [1 −1 0] direction, a_1_ is 0.22 nm experimentally). In some regions local p(2 × 2) order emerges, as in Fig. 4b (ovals).

**Figure 5:**
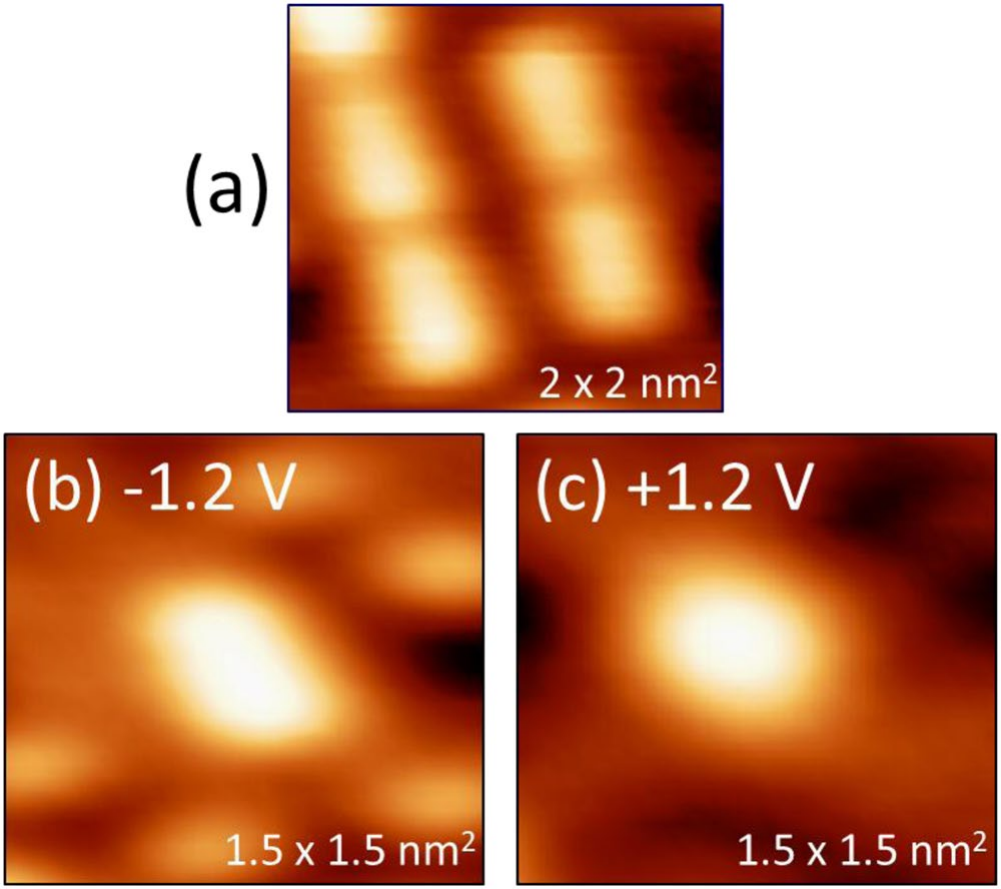
Bias dependence of the shape of the bright features, identified as AgS_2_ complexes. Tunneling conditions (*I, V*_*S*_) are (**a**) 1.0 nA, −1.0 V; (**b**) 0.9 nA, −1.2 V, (**c**) 0.9 nA, −1.2 V.

The second feature existing at this coverage is brighter and larger than a S adatom. Two examples are encircled in Fig. 4c. It is common to find these features grouped into chains, 2–7 units long, along the [1 −1 0] direction. Their bias dependence clearly distinguishes these features from S adatoms. As shown in Fig. 3c, the height of the bright features does not change with *V*_*S*_, remaining constant at 0.025 ± 0.003 nm, in contrast to the behavior of the S adatom features shown in Fig. 3b. The bright features do show a more subtle bias dependence, such that as the sign of *V*_*S*_ is switched, the shape changes from being oval-shaped, elongated in the [1 −1 0] direction, to being more circular. This can be seen in Fig. 5, and also Fig. 3a. The FWHM of the complexes at negative *V*_*S*_ is 0.63 ± 0.03 nm in the [1 −1 0] direction and 0.43 ± 0.02 nm in the [0 0 1] direction. The FWHM bears out the rounder nature of the complexes at positive *V*_*S*_, where the two values are 0.46 ± 0.03 nm, and 0.48 ± 0.03 nm, respectively. Even the smallest of these values is significantly larger than the FWHM of an S adatom, 0.31 ± 0.04 nm.

In addition, the bright features exhibit different short-range order. The separation within the bright chains is 0.66 ± 0.03 nm, which is 3a_1_, rather than the 2a_1_ spacing that is common between S adatoms.

Finally, the bright chains are often collinear with chains of S adatoms. This indicates that the bright features are centered above the troughs, like the S adatoms in Fig. 2a.

To summarize, the bright features differ from S adatoms in three major respects: Size, response to *V*_*S*_, and spacing within chains. Based on the following information, these bright features are identified as AgS_2_ complexes.

### Identification of AgS_2_ complexes from DFT

All DFT calculations described below were done with the PBE functional unless noted otherwise.

**Figure 6:**
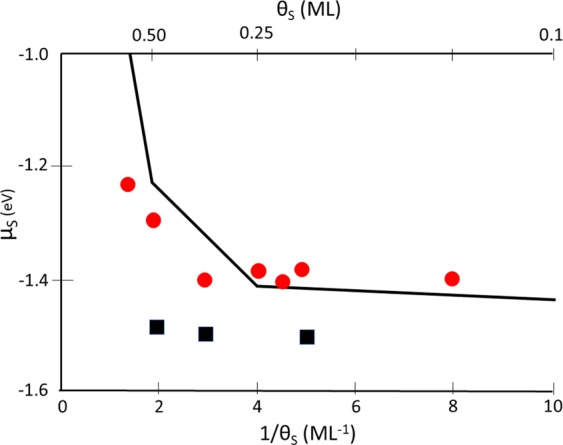
Coverage dependence of the chemical potential of S adatoms and S complexes on unreconstructed Ag(110). The solid line segments comprise the convex hull for the chemisorbed phases. Red dots show *µ*_*S*_ of most-stable AgS_2_ complexes in two-fold hollow sites, like those in Fig. 7. Black squares show most-stable zigzag configurations, like those in Fig. 9.

We have calculated the chemical potential of sulfur, *μ*_*S*_, for many configurations of S and Ag atoms on the Ag(110) surface. It is convenient to show the results as *μ*_*S*_ vs. 1/*θ*_*S*_, as in Fig. 6. With this choice of axes, we connect selected phases of the system by linear segments to form a convex hull of chemisorbed phases. More detail about the construction of the convex hull is given in the SI. This *μ*_*S*_ vs. 1/*θ*_*S*_ construction allows the chemisorbed phase composition at any coverage to be predicted from the well-known lever rule of thermodynamics, i.e. the solid lines can be treated as tie-lines. The main phases at *θ*_*S*_ ≤ 0.5 are the c(2 × 2) with *θ*_*S*_ = 0.5, and the p(2 × 2) with *θ*_*S*_ = 0.25. It can be seen that the coverage dependence of the adsorption energy is very small (<0.02 eV), for *θ*_*S*_ ≤ 0.25. The stability of the p(2 × 2) phase is consistent with the STM observations above.

**Figure 7:**
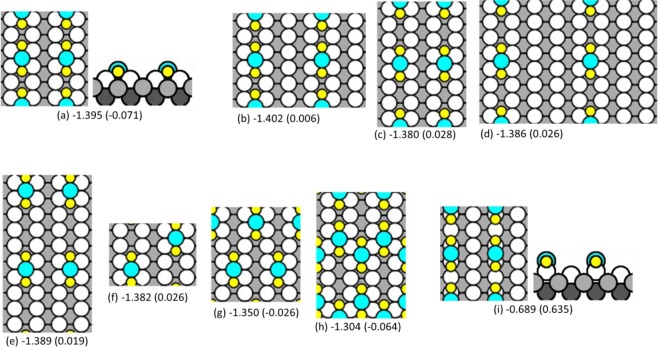
Schematics of AgS_2_ complexes with multiple configurations and coverages. In panels (a–h), AgS_2_ complexes reside within the trough as seen in side view (a), and occupy two fold hollow sites as shown in the top-down view. In panel (i), AgS_2_ resides on top of substrate rows as seen in side view, and occupies short bridge sites as shown in the top-down view. Beneath each configuration is the chemical potential of sulfur (*µ*_*S*_), with the chemical potential difference of the complex relative to the S adatom baseline (*∆µ*_*S*_) in parentheses. Coverages, supercell vectors, chemical potentials, and slab thicknesses are given in Supplementary Information (Table SI-2). Yellow circles are sulfur atoms, blue circles are additional Ag atoms, white circles are topmost atoms of the Ag(110) substrate, and gray circles are Ag atoms in lower layers (darker gray indicates a deeper location).

DFT also reveals the nature of the bright features. Among the complexes and reconstructions considered, the best fit to the experimental data is given by AgS_2_ complexes, where each complex is linear and aligned with the [1 −1 0] direction. The most stable configuration of such a complex has the Ag atom in each AgS_2_ unit above a two-fold hollow site and separated by 3a_1_ from its neighbor, as shown in Fig. 7a,b. The separation of 3a_1_ agrees exactly with experiment. Furthermore, the most stable phases have the AgS_2_ complexes arranged in simple rectangular patterns, rather than staggered. This also fits the experimental observation. The chemical potentials of such simple AgS_2_ phases are shown by the red circles in Fig. 6, relative to the baseline of chemisorbed phases. At low coverage, the AgS_2_ phase is slightly *less* stable than the S adatom phase, but close to *θ*_*S*_ = 0.25, it crosses over and becomes *more* stable than the S adatom phase. This is due mainly to variation in *μ*_*S*_ for the S adatom phases, since values for AgS_2_ phases are nearly constant. This crossover also agrees very well with experiment, which shows coexistence of these two phases at *θ*_*S*_ = 0.25. At higher coverage, *µ*_*S*_ of both AgS_2_ and chemisorbed phases rises sharply, but the value for AgS_2_ remains below the chemisorbed baseline, predicting that AgS_2_ complexes should be observed. We caution, however, that reconstructions may also become competitive at higher coverages.

**Figure 8:**
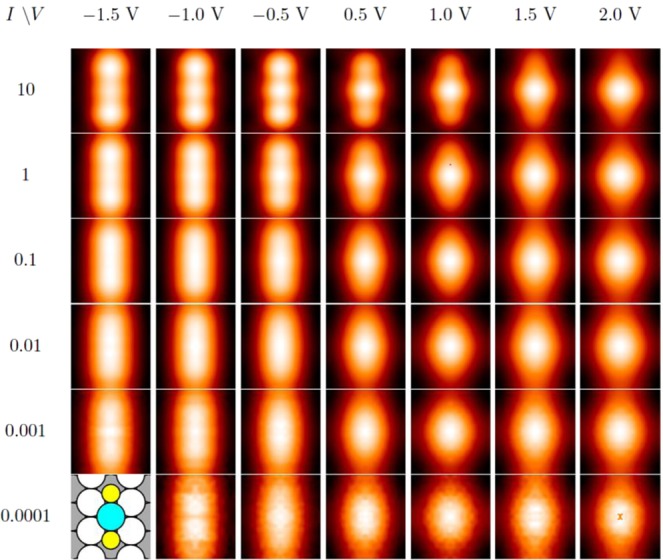
Bias dependence of AgS_2_ complexes, simulated using DFT for a (3 × 2) supercell with AgS_2_ complexes at the two-fold hollow site, and using PBE. Results are averaged over Ag(110) slab thicknesses *L* = 7 to 12. The schematic in the lower left shows the corresponding atom positions. Each column represents a fixed bias voltage *V*_*S*_, and each row represents fixed tunneling current *I*, in arbitrary units.

Further evidence comes from examining the bias dependence using DFT. Results are shown in Fig. 8 for different values of *I* and *V*_*S*_. As noted in Sec. 2, a value of *I* $\cong$ 0.001 a.u. should correspond to realistic experimental conditions. Two conclusions can be drawn from Fig. 8. First, the AgS_2_ complex appears as a protrusion under all conditions, consistent with experimental data (Fig. 3c). The apparent height of the complex ranges from 0.077 to 0.055 nm as *V*_*S*_ increases from −1.5 to 2.0 V. Second, the shape is elongated at negative voltage, but becomes rounder as bias voltage increases. This also agrees with experimental data (Fig. 3a and Fig. 5b,c).

In summary, the stability, periodicity, and bias dependence in STM images all confirm that the bright features are AgS_2_ complexes. It is noteworthy that these are discrete complexes, i.e. they do not share Ag or S atoms. Their discreteness is also demonstrated by the fact that individual complexes exist that are not part of a chain, as in the circles of Fig. 4c.

### Stability of other phases from DFT

**Figure 9:**
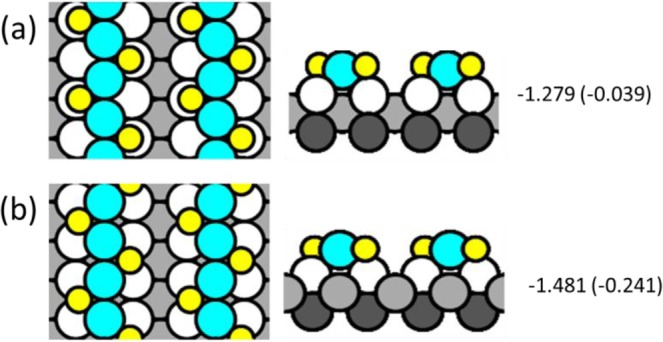
Top and side views of two possible configurations of zigzag Ag-S chains. Values of *µ*_*S*_ (in eV) are shown, calculated from DFT using PBE. The value in parentheses is the difference from baseline, (*∆µ*_*S*_). (**a**) Top rows of Ag atoms are in two-fold hollow sites with S adatoms in quasi-three-fold hollow sites. (**b**) Top row of Ag atoms occupy long bridge sites with S adatoms in quasi-four-fold hollow sites.

Some other phases are also quite stable, from DFT. Most notable are those where zigzag chains of S adatoms decorate the sides of linear rows of Ag atoms. Two simple examples of zigzag chains are shown in Fig. 9a,b. These are fundamentally different than the linear rows of AgS_2_ complexes shown in Fig. 7, since in the former case the AgS_2_ complexes are independent, whereas in the latter case the zigzag chains consist of concatenated AgS_2_ units, i.e. AgS_2_ units sharing S atoms. Many more zigzag configurations have been considered. Illustrations and details of these DFT calculations are given in Table SI-3. The two structures in Fig. 9a,b are similar except that in Fig. 9a, top rows of Ag atoms are in two-fold hollow sites, i.e. bulk-terminated sites. Consequently, S adatoms are in quasi-three-fold hollow sites. In Fig. 9b, top rows of Ag atoms occupy long bridge sites, which puts S adatoms in quasi-four-fold hollow sites. Despite the less-favorable coordination of the Ag atom, *µ*_*S*_ is much lower for the latter configuration than the former. This must be due to the more favorable bonding of S at a four-fold hollow site, which is well-known^54,55^. The most stable zigzag phases all have Ag atoms in long bridge sites and S adatoms in quasi-four-fold hollow sites. Values of *μ*_*S*_ for the most stable zigzag phases are shown as black squares in Fig. 6. They are significantly more stable than the adatom phases or the AgS_2_ complex phases. However, they all have periodicity 2a_1_, which precludes them from consideration based on the experimental data.

**Figure 10:**
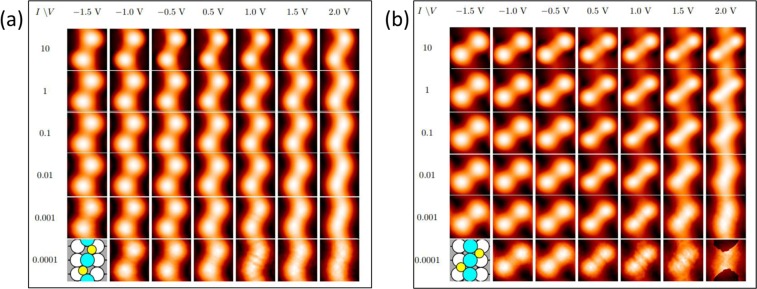
(**a**) DFT simulated STM images of a zigzag Ag-S chain configuration with a periodicity of 3a_1_. Schematic of configuration is shown in insert. (**b**) DFT simulated STM images of a different zigzag Ag-S chain configuration with a periodicity of 3a_1_. (**a**,**b**) configurations, coverages, supercell vectors, chemical potentials and slab thickness are given in Table SI-3 as Figure VI and Figure VII respectively.

Zigzag chains with a periodicity of 3a_1_ are also considered because that is the periodicity seen in the experimental data. Schematics are shown as insets in Fig. 10 (Larger schematics are shown as Fig. VI and Fig. VII in SI). These zigzag structures of Fig. 10a,b are significantly *less* stable than the discrete AgS_2_ complex phase (by 0.13 eV and 0.08 eV respectively, at fixed coverage of 0.33 ML using PBE). The zigzag structure of Fig. 10b is metastable and reconstructs at a larger slab thickness. The factor that mainly excludes these zigzag chains is the DFT simulated STM images (Fig. 10). The DFT simulated STM images of these zigzag chains do not match the shape and bias dependence of the bright features observed in experiments.

### Consideration of the functional in DFT

The absence of zigzag chains in experiment, yet their significantly higher stability in DFT (relative to the observed species, discrete AgS_2_ complexes), poses a quandary. On one hand, one might argue that the DFT predicts equilibrium phases, and perhaps the surface is not equilibrated in experiment. In particular, phases which incorporate extra Ag adatoms (as do the zigzag phases) might be blocked by energetic barriers. We reject this argument, however, on the basis that the discrete AgS_2_ species also involves incorporation of extra Ag, and this species is clearly observed. The other possibility is that the DFT is flawed. To examine this more carefully we perform additional DFT calculations using other functionals: RPBE,^56^ optB88-vdW^57,58^, meta-GGA SCAN^59^, and SCAN + rVV10^60^ functionals [Table 1]. Since all three structures–p(2 × 2) of S adatoms, AgS_2_ complexes, zigzag Ag-S chains–are compared at the same sulfur coverage of 0.25 ML S in Table 1, we can directly compare their relative stabilities using the difference in chemical potential (*∆µ*_*S*_) relative to the (2 × 2) phase. For discrete AgS_2_ complexes, all functionals predict a positive *∆µ*_*S*_, ranging from 0.01 to 0.05 eV. There is much wider variation in *∆µ*_*S*_ for the zigzag chains, where it ranges from −0.2 to +0.02 eV (although in no case is the zigzag chain predicted to be less stable than AgS_2_ complexes, i.e. no functional gives a result that is compatible with experiment). This wider variation in *∆µ*_*S*_ indicates that there are relatively large errors in DFT at the GGA level for the prediction of zigzag structure stability, which could explain the absence of zigzag structures in experimental observations.

**Table 1:** Comparison of various structures at 0.25 ML of sulfur, using different exchange-correlation approximations in DFT calculations.

	(2 × 2)-S	AgS_2_	Zigzag Ag-S Chains	*∆µ*_*S*_ (AgS_2_)	*∆µ*_*S*_ (Zigzag Ag-S chains)
PBE	−1.408	−1.380	−1.489	0.028	−0.081
RPBE	−1.119	−1.112	−1.298	0.007	−0.178
optB88-vdw	−1.623	−1.587	−1.635	0.036	−0.012
SCAN	−1.614	−1.574	−1.629	0.040	−0.015
SCAN + rVV10	−1.774	−1.722	−1.752	0.053	0.022

All numbers are chemical potentials of sulfur in units of eV. *∆µ*_*S*_ (X) is the different in chemical potentials for S between structure X and the (2 × 2)-S structure. A negative value indicates that it is more stable than the (2 × 2)-S structure. Results are obtained from averaging calculations with *L* = 7 to 12.

## Discussion

In this paper we have reported experimental and theoretical evidence of a discrete AgS_2_ complex on Ag(110). These complexes coexist with atomic sulfur at 0.25 monolayers, but they are very distinctive from atomic sulfur.

It is interesting to compare these observations with others introduced in Sec. 1. First, as noted there, we have previously identified sulfur-metal complexes on surfaces of Ag(111), Cu(111), and Au(100), at extremely low sulfur coverages (<0.01 ML). The present observation is distinctive in the sense that it is the first observation of the independent MS_2_ complex. In the previous studies, the MS_2_ subunit was identified as a component of the larger complex, but it was not observed independently.

Second, O/Ag(110) and S/Ag(110) systems show some similarities but also differences. The obvious similarity is the report of AgO_2_ and AgS_2_ complexes. However, a major difference is in the source of the Ag atoms. In AgO_2_, the source is the Ag atoms in the Ag rows, and formation of the complex leads to vacancies in the rows. In the present work, the Ag rows are unperturbed and the Ag atoms in the AgS_2_ complex must come from the two-dimensional Ag adatom gas (and, ultimately, from the Ag step edges). Another difference is in the experimental ability to isolate the discrete complex (unattached to other complexes or embedded in Ag rows). The discrete AgO_2_ complex was not observed directly (though its existence was inferred from the observation of Ag vacancies and the development of the added-row reconstruction.) Instead, the complex was only observed directly in its embedded form, wherein it can be regarded as two chemisorbed oxygen atoms adjacent to a somewhat-displaced Ag atom within a Ag row. In the present work, the AgS_2_ complex can be isolated and observed. The AgS_2_ complex is not embedded but resides above the surface plane within the troughs of the substrate (Fig. 7). A final point of comparison arises from the zig-zag chains. These are clearly observed in the O/Ag(110) system, again embedded within the surface, where they can be regarded as oxygen atoms densely decorating the sides of pre-existing Ag rows. We do not observe such features in the S/Ag(110) system. Despite these differences, the common evidence that AgO_2_ and AgS_2_ complexes can form on the Ag(110) surface is very interesting, and may have broader ramifications, especially for interpreting mass transport. Specifically, it has been observed that both oxygen and sulfur can strongly accelerate coarsening on many coinage metal surfaces^26–31^. While M_3_S_3_ is a strong candidate on the (111) surfaces, MS_2_ and MO_2_ are reasonable candidates for (110) and (100) surfaces. Indeed, AgS_2_ was implicated (but never observed directly) in enhanced coarsening on Ag(100)^25^. The present work, in which AgS_2_ is observed directly, lends credence to its existence on other surfaces.

Finally, it is interesting to comment on the strengths and weaknesses of the PBE functional in DFT as revealed by the present analysis. Considering only the discrete AgS_2_ complex phase and the chemisorbed phases, DFT does remarkably well. It predicts that the chemisorbed phase alone will be observed with increasing coverage until 0.25 ML, and then the complex phase will emerge. This is exactly what is observed in the experiment. However, DFT with PBE breaks down when considering the relative stability of the concatenated zigzag phases; it predicts that these should be observed in experiment even at very low coverages (Fig. 6)—displacing the chemisorbed phases–but the zigzag structure is not observed at all. The relative stability of the zigzag phases is more sensitive to the functional than is the relative stability of the discrete complex phase, suggesting that DFT does not treat the zigzag phases accurately at the level of PBE. The reasons for this are not fully understood but may be related to underestimation of the Ag bulk cohesive energy in PBE.

## Conclusion

In summary, adsorbed sulfur has been explored on Ag(110) surface at two different coverages, 0.02 and 0.25 ML. At 0.02 ML S, only sulfur adatoms are present. At 0.25 ML S, there is coexistence of sulfur adatoms and bright features. Sulfur adatoms exhibit local ordering in the form of short chains with sulfur adatoms separated by 2a_1_, or small local p(2 × 2). The bright features also form short chains but are separated by 3a_1_. These bright features are determined to be linear AgS_2_ complexes from DFT. Experimental observations, such as bias dependence and separation of complexes, agree well with DFT prediction. DFT also predicts the absence of AgS_2_ complexes at 0.02 ML, and the coexistence of S adatoms and AgS_2_ complexes at 0.25 ML of sulfur, which is experimentally observed. Other phases are determined to be more stable than S adatoms and AgS_2_ complexes, mainly zigzag Ag-S chains with a periodicity of 2a_1_, but are not considered to be the bright features due to periodicity mismatch. To our knowledge, this is the first example of an isolated linear MS_2_ complex.

## Supplementary information

Supplementary information

## Data Availability

STM images and data measurements can be obtained at 10.25380/iastate.9763058.
