# Supplementary material for: Identification of an AgS2 Complex on Ag(110)
Source: Sci Rep. 2019 Dec 27;9:19842. doi: 10.1038/s41598-019-56275-4 (PMC6934528; doi:10.1038/s41598-019-56275-4)

**Supplemental Information for *Identification of an AgS_2_ Complex on Ag(110)***

Peter M. Spurgeon,^a,^* Da-Jiang Liu,^c^ Junepyo Oh,^b^ Yousoo Kim,^b^ and Patricia A. Thiel^a,c,d^

^a^Department of Chemistry, Iowa State University, Ames, Iowa 50011 USA

^b^ RIKEN Surface and Interface Science Laboratory, Wako, Saitama 351-0198, Japan

^c^ Ames Laboratory of the USDOE, Ames, Iowa 50011 USA

^d^Department of Materials Science and Engineering, Iowa State University, Ames, Iowa 50011 USA

* peterms@iastate.edu (515) 294-0905

**Contents of the Supplemental Information**

**I. Details about construction of the energy baseline for chemisorbed phases.**

**II. Table SI-1. Details about configurations and DFT calculations for chemisorbed phases (S adatom phases) on the unreconstructed Ag(110) surface.**

**III. Table SI-2. Details about configurations and DFT calculations for AgS_2_ complexes, represented in Fig. 7 of the main text.**

**IV. Table SI-3. Configurations and computational details for zigzag Ag-S chains.**

**V. Schematic representations of other configurations tested, including values of *µ_S_* with PBE.**

**I. Details about construction of the energy baseline for chemisorbed phases.**

The baseline for chemisorbed phases is constructed as shown in Fig. SI-1.

**
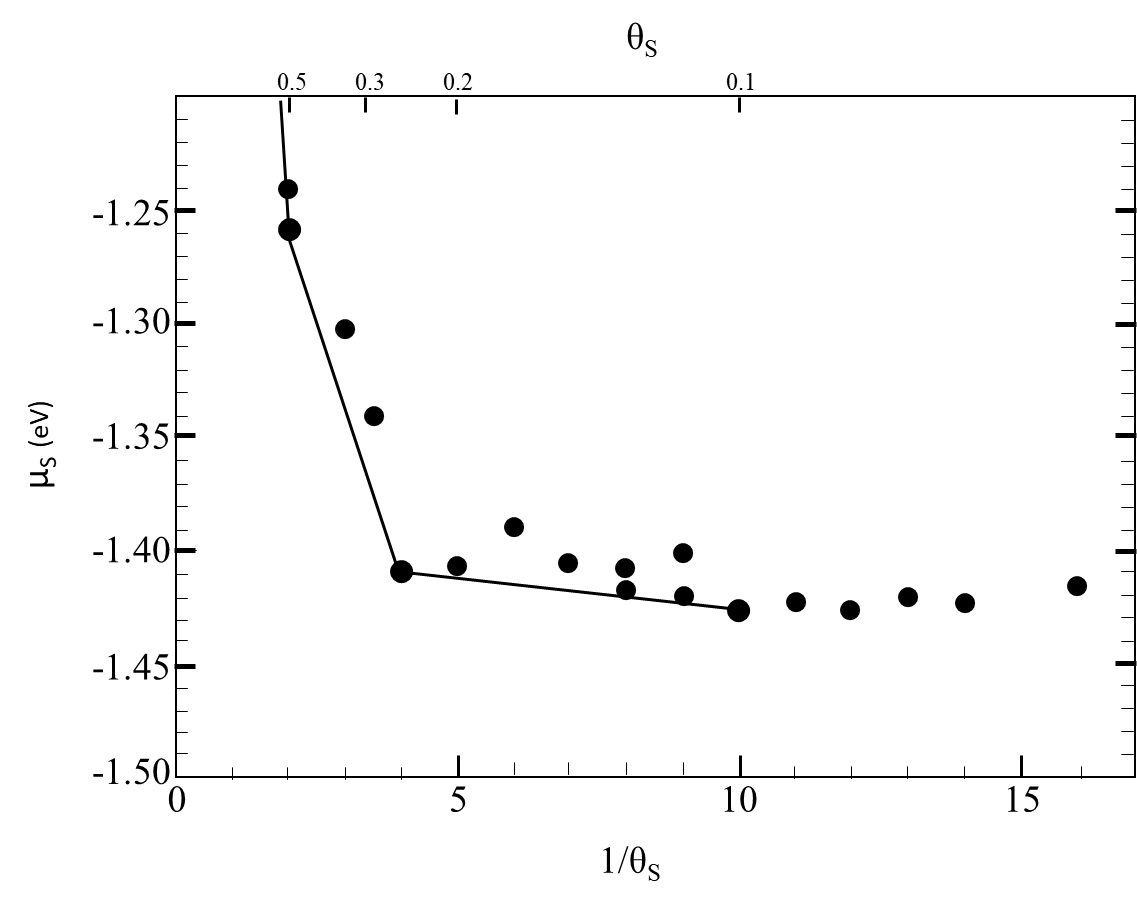
**

**Figure SI-1.** Coverage dependence of the chemical potential (adsorption energy) of S adatoms on unreconstructed Ag(110). The solid line connects the convex hull with θ_S_ = 1, 0.5, 0.25, 0.10 ML, which are highlighted by boldface in Table SI-1. Values of *µ_S_* are calculated using PBE.

**II.** **Table SI-1.** Details about configurations and DFT calculations for chemisorbed phases (S adatom phases) on the unreconstructed Ag(110) surface. *b1* and *b2* are supercell vectors, expressed in terms of basis vectors *a1* = (-1/2, 1/2, 0) and *a2* = (0, 0, 1). *μ_S_^e^* is the numerical uncertainty in *μ_S_*, obtained from the standard deviations of *μ_S_* for different slab thickness *L*, divided by the number of slabs used in the averaging, with range [*L_min_, L_max_*].

| (*b_1_, b_2_*) | θ_S_ | *μ_S_* (eV) | *μ_S_^e^* | Δ *μ_S_* | *L_min_, L_max_* |
| --- | --- | --- | --- | --- | --- |
| **((1 0), (0 -1))** | **1.00** | **-0.7660** | **0.0014** | **0.0000** | **7, 12** |
| **((0 1), (2 0))** | **0.50** | **-1.2550** | **0.0009** | **0.0000** | **7, 12** |
| ((-1 1), (1 1)) | 0.50 | -1.2404 | 0.0047 | 0.0146 | 7, 12 |
| ((1 -1), (2 1)) | 0.33 | -1.3103 | 0.0022 | 0.0218 | 7, 12 |
| ((-1 -2), (-3 1)) | 0.29 | -1.3421 | 0.0019 | 0.0286 | 7, 12 |
| **((2 1), (-2 1))** | **0.25** | **-1.4093** | **0.0024** | **0.0000** | **7, 12** |
| ((0 2), (2 0)) | 0.25 | -1.4078 | 0.0035 | 0.0015 | 7, 12 |
| ((1 2), (2 -1)) | 0.20 | -1.4055 | 0.0027 | 0.0066 | 7, 12 |
| ((0 2), (3 0)) | 0.17 | -1.3869 | 0.0038 | 0.0280 | 7, 12 |
| ((-1 -2), (-3 1)) | 0.14 | -1.4017 | 0.0025 | 0.0161 | 7, 12 |
| ((-3 1), (2 2)) | 0.13 | -1.4193 | 0.0030 | 0.0013 | 7, 12 |
| ((-2 2), (2 2)) | 0.13 | -1.4121 | 0.0031 | 0.0085 | 7, 12 |
| ((0 3), (3 0)) | 0.11 | -1.4016 | 0.0040 | 0.0219 | 7, 12 |
| ((-3 1), (3 2)) | 0.11 | -1.4218 | 0.0032 | 0.0017 | 7, 12 |
| **((2 2), (-3 2))** | **0.10** | **-1.4263** | **0.0039** | **0.0000** | **7, 12** |
| ((-3 2), (4 1)) | 0.09 | -1.4226 | 0.0048 | 0.0065 | 7, 12 |
| ((-3 2), (3 2)) | 0.08 | -1.4252 | 0.0065 | 0.0068 | 7, 12 |
| ((-4 1), (1 3)) | 0.08 | -1.4184 | 0.0041 | 0.0164 | 7, 12 |
| ((-4 1), (2 3)) | 0.07 | -1.4226 | 0.0021 | 0.0151 | 7, 12 |
| ((0 4), (4 0)) | 0.06 | -1.4158 | 0.0040 | 0.0276 | 7, 12 |

**III. Table SI-2.** Details about configurations and DFT calculations for AgS_2_ complexes, represented in Fig. 7 of the main text. *b1* and *b2* are supercell vectors, expressed in terms of basis vectors *a1* = (-1/2, 1/2, 0) and *a2* = (0, 0, 1). *μ_S_^e^* is the numerical uncertainty in *μ_S_*, obtained from the standard deviations of *μ_S_* for different slab thickness *L*, divided by the number of slabs used in the averaging, with range [*L_min_, L_max_*].

| Fig. | (*b_1_, b_2_*) | θ_S_ | *μ_S_* (eV) | *μ_S_^e^* | Δ *μ_S_* | *L_min_, L_max_* |
| --- | --- | --- | --- | --- | --- | --- |
| 7a | ((0 2), (3 0)) | 0.33 | -1.3946 | 0.0016 | -0.0625 | 7, 12 |
| 7b | ((0 3), (3 0)) | 0.22 | -1.4023 | 0.0019 | 0.0084 | 7, 12 |
| 7c | ((0 2), (4 0)) | 0.25 | -1.3800 | 0.0017 | 0.0292 | 7, 12 |
| 7d | ((0 4), (4 0)) | 0.13 | -1.3863 | 0.0078 | 0.0343 | 4, 7 |
| 7e | ((0 2), (5 0)) | 0.20 | -1.3894 | 0.0018 | 0.0194 | 7, 12 |
| 7f | ((-2 2), (2 2)) | 0.25 | -1.3821 | 0.0034 | 0.0271 | 7, 12 |
| 7g | ((0 2), (3 1)) | 0.33 | -1.3500 | 0.0063 | -0.0179 | 4, 7 |
| 7h | ((0 2), (4 1)) | 0.50 | -1.3060 | 0.0070 | -0.0510 | 4, 7 |
| 7i | ((0 2), (3 0)) | 0.33 | -0.6888 | 0.0179 | 0.6433 | 4, 7 |

**III. Table SI-3.** Configurations and computational details for zigzag Ag-S chains. Notation is same as in Table SI-1.

| Fig. | (*b_1_, b_2_*) | θ_S_ | *μ_S_* (eV) | *μ_S_^e^* | Δ *μ_S_* | *L_min_, L_max_* |
| --- | --- | --- | --- | --- | --- | --- |
| I | ((0 2), (2 0)) | 0.50 | -1.2791 | 0.0273 | -0.0241 | 4, 7 |
| II | ((0 2), (2 0)) | 0.50 | -1.4812 | 0.0057 | -0.2263 | 7, 12 |
| III | ((0 3), (2 0)) | 0.33 | -1.4880 | 0.0048 | -0.1559 | 7, 12 |
| IV | ((0 4), (2 0)) | 0.25 | -1.4891 | 0.0053 | -0.0798 | 7, 12 |
| V | ((0 5), (2 0) | 0.20 | -1.4904 | 0.0043 | -0.0783 | 7, 12 |
| VI | ((0 2), (3 0)) | 0.33 | -1.2669 | 0.0054 | 0.0652 | 7, 12 |
| VII | ((0 2), (3 0)) | 0.33 | -1.4089 | 0.0064 | -0.0768 | 7, 12 |


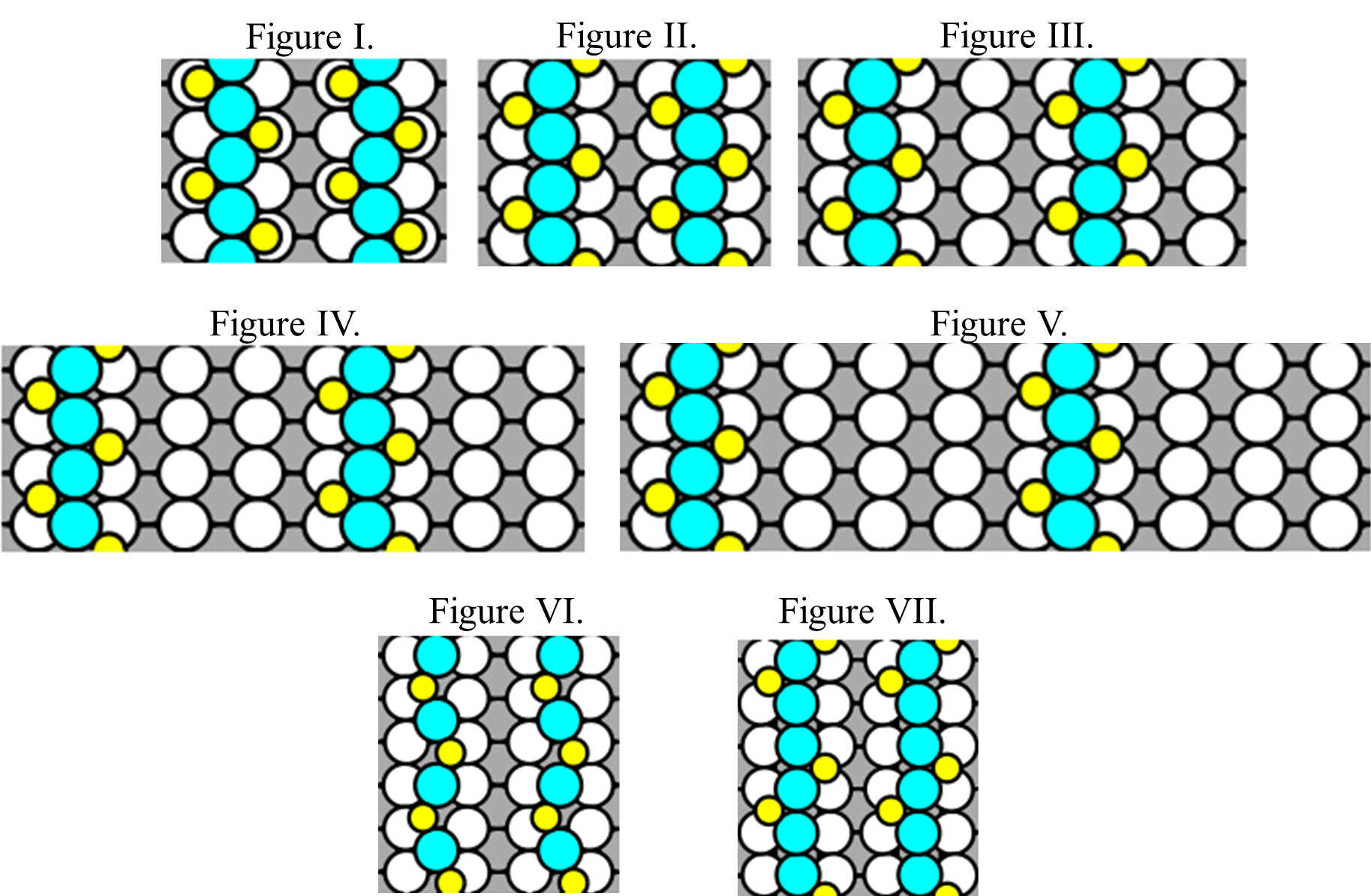


Fig. I and Fig. II are the same configuration as Figure 9a and 9b in text respectively. DFT simulated STM images of Fig. 10a and Fig. 10b in the text correspond to the configurations of Fig. VI and Fig. VII respectively.

**IV. Schematic representations of other configurations tested, including values of *µ_S_* with PBE.**

| Fig. | (*b_1_, b_2_*) | θ_S_ | *μ_S_* (eV) | *μ_S_^e^* | Δ *μ_S_* | *L_min_, L_max_* |
| --- | --- | --- | --- | --- | --- | --- |
| a | ((0 2), (3 0)) | 0.33 | -1.3371 | 0.0032 | -0.0050 | 7, 12 |
| b | ((0 3), (2 0)) | 0.33 | -1.3430 | 0.0024 | -0.0109 | 7, 12 |
| c | ((0 3), (3 0)) | 0.22 | -1.3719 | 0.0089 | 0.0388 | 7, 12 |
| d | ((0 1), (2 0)) | 0.50 | -0.8020 | 0.0054 | 0.4529 | 7, 12 |
| e | ((0 -1), (-3 0)) | 0.67 | -1.2395 | 0.0066 | -0.2290 | 4, 7 |
| f | ((-1 1), (3 0)) | 0.67 | -1.2900 | 0.0077 | -0.2795 | 4, 7 |
| g | ((0 2), (2 0)) | 0.50 | -1.1624 | 0.0147 | 0.0925 | 4, 7 |
| h | ((0 2), (2 0)) | 0.50 | -0.9785 | 0.0113 | 0.2764 | 4, 7 |
| i | ((0 2), (2 0)) | 0.50 | -1.1674 | 0.0084 | 0.0876 | 4, 7 |
| j | ((1 2), (3 0)) | 0.33 | -1.3904 | 0.0090 | -0.0583 | 4, 6 |
| k | ((0 2), (3 0)) | 0.50 | -1.2961 | 0.0084 | -0.0412 | 4, 7 |
| l | ((0 -1), (-3 0)) | 0.67 | -1.1197 | 0.0075 | -0.1092 | 7, 12 |
| m | ((0 2), (2 0)) | 0.50 | -1.4812 | 0.0057 | -0.2263 | 7, 12 |
| n | ((0 2), (2 0)) | 0.50 | -1.2791 | 0.0273 | -0.0241 | 4, 7 |
| o | ((1 2), (2 0)) | 0.50 | -1.4669 | 0.0204 | -0.2119 | 4, 7 |
| p | ((0 3), (2 0)) | 0.33 | -1.4880 | 0.0048 | -0.1559 | 7, 12 |
| q | ((0 4), (2 0)) | 0.25 | -1.4891 | 0.0053 | -0.0798 | 7, 12 |
| r | ((1 2), (2 0)) | 0.75 | -1.2353 | 0.0142 | -0.3064 | 4, 7 |
| s | ((0 2), (4 1)) | 0.50 | -1.3060 | 0.0070 | -0.0510 | 4, 7 |
| t | ((0 3), (3 0)) | 0.44 | -1.3519 | 0.0059 | -0.0777 | 4, 7 |
| u | ((0 3), (2 0)) | 0.33 | -1.3926 | 0.0304 | -0.0605 | 4, 7 |
| v | ((0 4), (2 0)) | 0.50 | -1.4376 | 0.0064 | -0.1827 | 7, 12 |
| w | ((0 2), (2 1)) | 0.25 | -1.5040 | 0.0085 | -0.0947 | 7, 12 |
| x | ((0 3), (2 0)) | 0.33 | -1.2916 | 0.0142 | 0.0405 | 4, 7 |
| y | ((0 3), (2 0)) | 0.67 | -1.3693 | 0.0051 | -0.3588 | 6, 10 |
| z | ((0 3), (2 0)) | 0.67 | -1.2387 | 0.0116 | -0.2282 | 4, 7 |
| aa | ((0 3), (3 0)) | 0.44 | -1.3009 | 0.0042 | -0.0266 | 4, 7 |
| ab | ((0 4), (2 0)) | 0.63 | -1.2912 | 0.0125 | -0.2318 | 4, 7 |
| ac | ((1 3), (2 0)) | 0.83 | -1.0375 | 0.0037 | -0.1737 | 4, 7 |
| ad | ((0 4), (2 0)) | 0.75 | -0.9480 | 0.0055 | -0.0190 | 4, 7 |
| ae | ((1 4), (2 0)) | 0.88 | -1.0293 | 0.0096 | -0.1935 | 4, 7 |
| af | ((0 5), (2 0)) | 0.70 | -1.0459 | 0.0099 | -0.0704 | 4, 7 |
| ag | ((1 5), (2 0)) | 0.70 | -1.3130 | 0.0074 | -0.3375 | 4, 7 |
| ah | ((0 7), (2 0)) | 0.71 | -1.3152 | 0.0074 | -0.3375 | 4, 7 |
| ai | ((0 8), (2 0)) | 0.63 | -1.2916 | 0.0131 | -0.2322 | 4, 7 |
| aj | ((1 9), (2 0)) | 0.72 | -1.2929 | 0.0062 | -0.3389 | 4, 7 |


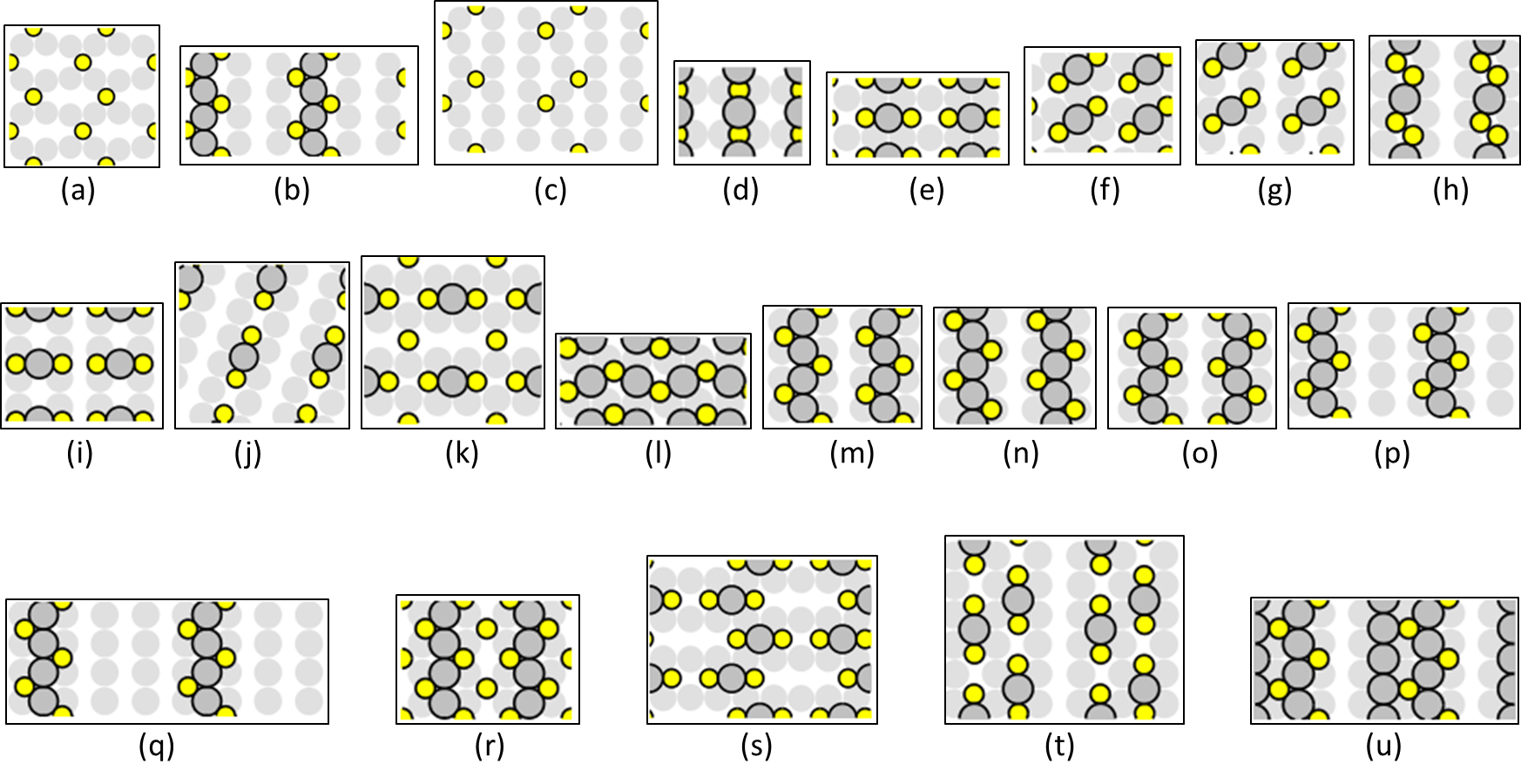


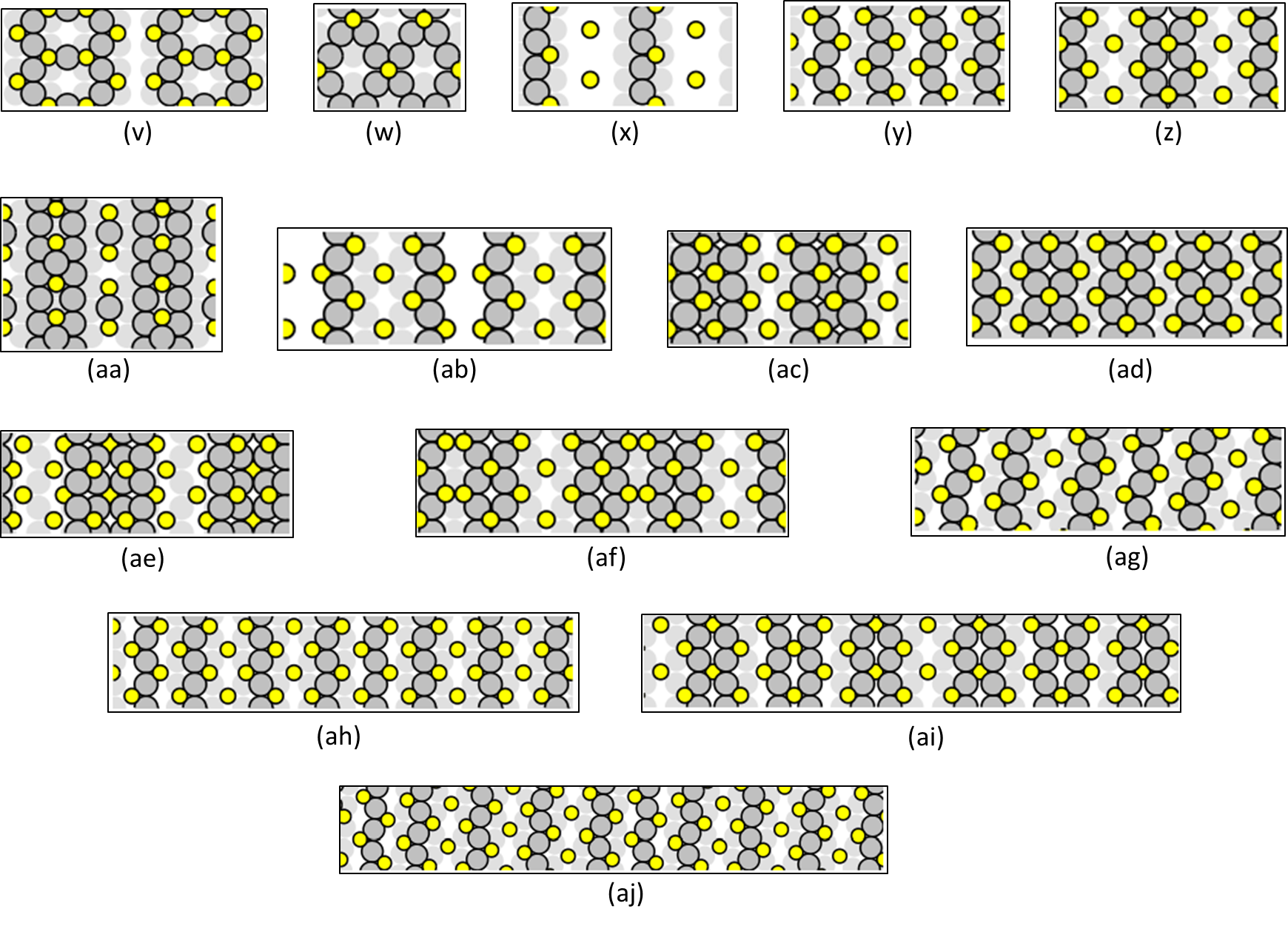

Supplement: Supplementary file 1 — Supplementary information [file 41598_2019_56275_MOESM1_ESM.docx]
